# Interventional oncology of liver tumors: how it all started and where are we now

**DOI:** 10.1259/bjr.20220434

**Published:** 2022-07-12

**Authors:** Luigi A. Solbiati, Yasuaki Arai

**Affiliations:** 1 Department of Biomedical Sciences, Humanitas University, Rozzano, Milan, Italy; 2 Department of Radiology, IRCCS Humanitas Research Hospital, Rozzano, Milan, Italy; 3 Department of Diagnostic Radiology, National Cancer Center, Tokyo, Japan

## Abstract

Liver was the very first organ for which interventional procedures were applied for the local treatment of primary and secondary malignancies. In this paper, the history of Interventional Oncology of liver, from the very beginning to the current situation, is summarized, including both percutaneous and intravascular procedures, and together with the evolution of the techniques for image guidance. The main ongoing developments, such as new techniques, combined interventional treatments and association of local interventions with new drugs are briefly described, too.

## How it all started

The term” Interventional Oncology” (IO) was introduced in 2006 at the first World Conference held in Cernobbio (Lake Como, Italy), replacing the previous definition of “image-guided therapies of oncologic diseases”. IO includes therapies directly applied that eradicate or substantially destroy focal tumors (ablations) and treatments applied via intravascular route. Ablations can be categorized into three major groups: (i) injectables (ethanol, acetic acid and hot saline), (ii) heating (radiofrequency, laser, microwaves and high-intensity focused ultrasound) and (iii) freezing (cryotherapy). Direct ablation and intravascular procedures aim to cure tumors without removal. They were born separately and at slightly different times, but nowadays are frequently used in “combination”.

Being easily approachable by imaging modalities and very frequent location of neoplastic diseases of various origin, the liver was historically the very first organ undergone IO procedures. Between the end of the 70’s and the beginning of the 80’s, the technical feasibility and the clinical role of direct percutaneous injection of toxic drugs were investigated for the treatment of liver malignancies. After previous *in vivo* research on animals, in 1983 Livraghi et al started a protocol of ultrasound-guided injection of various types of chemotherapeutic agents (according to tumor histology) into neoplasms of liver, pancreas, peritoneum and lung. The results were encouraging, safely achieving partial response in up to 60% of tumors previously undergone unsuccessful systemic chemotherapy.^1^ Previously, in February 1982, in a patient affected with primary hyperparathyroidism (PHPT), Solbiati et al biopsied a neck mass, causing a small intralesional hematoma. The diagnosis of parathyroid adenoma was confirmed, but unexpectedly associated with temporary decrease of parathormone level, likely due to the compression of functional tissue by the small intralesional hematoma caused by the biopsy. Given the comorbidities of this patient, it was thought to take the most of this advantage injecting percutaneously, with ultrasound guidance, a small amount (2.5 cc) of 95% ethyl alcohol (ethanol) in three consecutive sessions. The outcome was a permanent cure of the PHPT. The decision to inject alcohol as chemical agent, already clinically used for celiac nerve block^2^ and treatment of large renal and hepatic cysts,^3^ was based on its effects on tissues: immediate dehydration of cytoplasmic proteins with consequent coagulation necrosis followed by fibrosis and necrosis of endothelial cells, and platelet aggregation with thrombosis of small vessels followed by tissue ischemia. Additionally, the capsule of parathyroid adenomas allowed ethanol, slowly injected, to remain inside the nodule, avoiding diffusion into perilesional tissues. To our knowledge, this was the very first percutaneous “treatment without removal” of solid tumor ever performed and led to the publication of the first paper worldwide on local treatment of solid tumors by percutaneous ethanol injection (PEI).^4^

Following this preliminary experience, several centers started performing PEI of hepatocellular carcinomas (HCCs) because, in addition to the hypervascularity and the presence of perilesional capsule, similar to parathyroid adenomas, HCCs had another peculiarity favouring PEI, *i.e.* the softer consistency compared to the surrounding hard cirrhotic liver, that allowed ethanol to easily and uniformly diffuse inside the neoplastic tissue.^5,6^ Being quickly and precisely performable and monitorable by ultrasound guidance, extremely cheap and safe, PEI rapidly spread particularly in countries where HCC was greatly diffused (South Europe, Japan and China), but with limited possibilities of local treatments.

Given that, at the beginning of the 80s, CT and MRI had already demonstrated the peculiar vascular characteristic of HCCs, *i.e*. the almost exclusive arterial supply compared with the predominantly portal supply of the whole liver, the intravascular approach to HCC without (transarterial embolization, TAE) or with anticancer drugs (transcatheter arterial chemoembolization, TACE) was attempted, with better results than those of systemic administration. In 1983, Yamada et al reported their preliminary results with TACE using an anticancer drug and gelatin sponge,^7^ and in the same year, Konno et al demonstrated that lipiodol accumulated in HCC when it was injected into the hepatic artery.^8^ TACE with emulsion of lipiodol, anticancer agent and gelatin sponge became one of standard focal treatments of HCC (conventional TACE or cTACE).^9,10^

In the meantime, in the first 90s the efficacy of PEI for HCC was demonstrated in several mono-^11,12^ and multicenter^13,14^ studies, with substantial comparability of survival between PEI and hepatic resection at 3–5 years. It was demonstrated that the greatest success of PEI was achievable for solitary tumors 3 cm or less in diameter, but also that ultrasound-guided single-session injection of large amounts of alcohol (up to 165 ml) in general anesthesia was feasible.^15^ Even surface lesions and those adjacent to large vessels could be treated safely and effectively, having only uncontrollable coagulopathy, ascites, and extrahepatic metastases as absolute contraindications. Mortality related to PEI was extremely rare, and major complications ranged from 1.3 to 2.4%.^16,17^ The treatment of neoplastic portal thrombosis with PEI was also attempted, but the long-term outcomes were not very successful.^18^ Differently from surgical resection, PEI could be repeated because repeat sessions did not compromise hepatic function.^13,17^ The most relevant cause of local tumor progression (LTP) was the inability of alcohol to treat HCC satellites because ethanol had to remain inside the lesions to be effective.

Absence of peripheral capsule, harder consistency compared to the surrounding liver causing greater resistance to ethanol diffusion, and poor vascularity were the most important causes of unsuccessful results of PEI for the treatment of liver metastases,^19^ with complete response achieved most often for lesions smaller than 2 cm in size and for metastases from neuroendocrine carcinomas, usually hypervascular like HCCs.

In 1994, Ohnishi et al described in a single report^20^ the use of 50% acetic acid solution injection as potential alternative to PEI for therapy of small HCCs. Despite the good results achieved, although with stronger local pain compared to PEI during the injection, this experience remained isolated and no further results were reported. In the same year (1994), percutaneous hot saline injection (PSIT) was proposed, in a single report,^21^ as an alternative to PEI for percutaneous HCC ablation. PSIT caused target destruction via heat-induced coagulation necrosis rather than protein denaturation. No major complications and only moderate burning pain during the injection, associated with transient fever were reported. As most patients were followed only for 1 year or less, no conclusions were drawn about survival.

In order to increase the size of volumes of necrosis achieved with PEI and to overcome the limitations of PEI for HCC, the very first “combined” treatments were introduced in 1990–1992, using PEI after previous transcatheter chemoembolization.^22,23^

In the same years, the use of local treatments no longer based on chemical effect, but on physical (thermal) action were explored, aiming to ablate also liver metastases in addition to HCCs. The first thermal technique clinically available was interstitial laser photocoagulation (ILP), initially described by Steger et al in 1989.^24^ The thermal coagulation effect was provided by the conversion of absorbed light, following scattering by tissue, into heat. Low absorption and high scattering of neodymium:yttrium-aluminum-garnet (Nd:YAG) laser maximized penetration causing cytotoxic effect. Although ILP was potentially useful also in the therapy of HCCs, it was predominantly used for liver metastases with ultrasound guidance^25,26^ or guided by thermosensitive MRI sequences.^27^ No major or minor complications were reported and the outcomes were satisfactory only for lesions measuring 20 mm or less. This fact, associated with the complex need to employ multiple fibers regularly spaced, actually caused the interruption of the clinical use of ILP for ablation of liver malignancies.

After extensive experimental studies in animal models,^28,29^ in 1993 Rossi et al^30^ and McGahan et al^31^ reported their first applications of radiofrequency ablation (RFA) in patients with small HCC. Radiofrequency (RF) waves induce ionic agitation in tissues, which results in frictional heat production, generated by means of impedance that the surrounding tissue opposes to the flow of current, so that heat is not generated at the tip of the electrode but within the tissue. The heat produced is given by the difference between the heat generated around the extremity of the electrode and the dispersed heat, whose entity depends on tissue conductivity and dissipation due to proximity to blood vessels (heat-sink effect). The final result is local tissue destruction by coagulation necrosis. The size and shape of the necrotic lesion produced are a function of probe gauge, length of the exposed probe tip, temperature along the exposed electrode, and duration of therapy.^32^ In 1995, the very first RF probes with internal cooling system were introduced in clinical practice. Solbiati et al and Livraghi et al, in collaboration with the Research Center of the Massachusetts General Hospital in Boston, started the first protocol of treatment with cool-tip electrodes of liver metastases.^33^ Subsequent improvements aiming at increasing the volumes of necrosis achievable, either technical (multiprobe or “cluster” arrays)^34,35^ or clinical (saline enhancement)^36^ were tested, but the increased risk of bleeding and uncontrollable diffusion of hot saline, respectively, took to the decision to stop these experimental trials. In the following years, several types (single cooled-tip, multitined, perfusion electrodes, etc..) and arrangements (monopolar, multipolar) of RF applicators were made available and clinically tested.

For the treatment of HCC, RFA showed better local efficacy and required fewer treatment sessions compared to PEI. Particularly, in tumors smaller than 3 cm in size, RFA obtained complete ablation in nearly the totality of cases, and very frequently an additional 0.5–1.0 cm safety margin around the HCCs, thus significantly reducing the number of LTPs on the follow-up.^37^ Also for HCCs of medium and large size RFA allowed to achieve results definitely better than those obtained with PEI, either alone^38^ or in combination with TAE.^39^

The history of cryoablation for the treatment of liver malignancies started in the late 80s as “cryosurgery”. Liquid nitrogen was initially placed directly on tissue intraoperatively, but the most significant development occurred when applicators to be introduced percutaneously or endocavitarily were made available. The quick application of cold initially results in the formation of intracellular ice crystals that have destructive effect on cell membrane and cell organelles. During thawing, water flows from the hypotonic interstitium into the cells causing further cell damage and bursting. In addition, ice forms also in the endothelium of vessels causing the collapse of the blood supply.^40^

The cytotoxic effect is precisely monitored and followed by imaging methods, with clear demarcation between frozen and normal tissue.^41^ Despite these interesting characteristics, cryoablation has been rarely applied in clinical practice for liver tumors^42,43^ because of the reported high incidence of severe complications (cryoshock and acute respiratory distress syndrome) and its high costs compared to other ablative modalities.

Microwave tissue coagulation was initially used to achieve hemostasis along incision planes in an effort to reduce blood loss during hepatic resection, and intraoperatively as an alternative to hepatectomy in patients with unresectable HCC.^44^ When needle-like microwave applicators (so-called “antennas”) became clinically available, microwave ablation (MWA) progressively developed as alternative to RFA for percutaneous treatment of liver malignancies.^45,46^ The 915 or 2450 MHz microwaves create a rapidly alternating electromagnetic field. Water molecules follow the changing polarity of the field and heat is generated from within the tissue, causing coagulation necrosis. Initially, microwave systems had low power, thus multiple antennas had to be often inserted to achieve sufficiently large volumes of necrosis.^45–47^ For this reason, for some years MWA was used only in some Asian countries where the cost of microwave systems was significantly lower than that of RFA systems. With the introduction of high-power MWA, the technical advantages of the microwaves compared to the RF systems became clear: capability of achieving larger ablation volumes in shorter operative times, more predictable shape of the ablation volume and significantly less heat sink effect.^48,49^ MWA is more effective than RFA for perivascular tumors, while for peribiliary tumors the complication rates are higher for MWA than for RFA.^50^

Irreversible electroporation (IRE) is the most novel ablation technique for liver malignancies.

Described for the first time by Davalos et al in 2005^51^ and clinically used for liver tumors in 2012,^52^ IRE is a non-thermal technique. Micro- to millisecond high-voltage electrical pulses are delivered by thin electrodes to induce permanent cell membrane permeability and complete hepatocyte cell death, with a narrow transition between the ablation zone and the surrounding completely uninjured tissue. IRE mandatorily requires time-consuming insertion of multiple electrodes regularly spaced around the target tumor. Consequently, stereotactic guidance has been shown particularly useful to reduce procedure length and improve electrode placement accuracy. Differently from all the other ablative techniques, IRE must be always performed under general anesthesia with complete muscle relaxation and ECG synchronization during delivery. Enabling to preserve from ablation all the connective tissue-rich structures (*e.g.* biliary duct and blood vessel walls), IRE is mostly used for liver tumors adjacent to anatomical structures “at risk”, like tumors at Segment 1 or proximal to major bile ducts and for tumor recurrences in small liver remnants.^53,54^ IRE does not use heat to eradicate tumors, thus its efficacy is not impeded by the heat-sink effect on adjacent blood vessels. Despite these favorable technical and clinical aspects, data concerning efficacy and safety of hepatic IRE are still scanty. In the systematic review by Scheffer et al including 16 studies,^55^ the efficacy was very variable likely because of the heterogeneity in size and origin of treated tumors, patient population, and operator experience (given the steep learning curve of IRE), and the overall complication rate was relatively low (16%). However, in a very recent paper^56^ dealing with IRE of small- and medium-sized unresectable colorectal liver metastases (CRLMs), repeat treatments had to be performed in 22% of patients with final local control in 74% of cases. The 1 year LTP-free survival was 68% and the median overall survival (OS) was 2.7 years from the first IRE (longer than reported in many surgical series although in nonsurgical candidates), but the overall complication rate was high (40%), including major adverse events (portal vein thrombosis, biliary complications, etc..) in 33.3% of patients and one death (2%). Consequently, further evaluation about efficacy and safety of hepatic IRE is needed.

In the field of intravascular therapies, in the last 15 years, the most relevant innovations have been transarterial radioembolization (TARE), super-selective TACE, DEB-TACE and DEBIRI-TACE.

The very first results achieved with TARE using resin or glass microspheres labeled with yttrium-90 (^90^Y) for the treatment of unresectable HCC^57^ and hepatic metastases^58^ were published in 1994 and 2002, respectively. Super-selective TACE, *i.e.* the selective insertion of microcatheters into the tumor feeding artery in order to maximize the effect on HCC and minimize it on normal liver, was initially developed in Japan,^59,60^ and then diffused to other Asian countries, also favored by the introduction of new imaging modalities, like angio-CT^61^ and cone beam CT. On the other hand, in Western countries, microspheres with drug-eluting (DEB) capabilities were developed and injected during cTACE. DEB-TACE, clinically used for the first time in 2007,^62^ allows for controlled and sustained drug delivery (doxorubicin hydrochloride) with minor dispersion of the drug compared with c-TACE. In 2008, the first paper on irinotecan-eluting beads (DEBIRI-TACE) for the treatment of CRLMs as palliative setting was published.^63^

As regards the techniques for image guidance of percutaneous ablations, since the beginning of the history of IO ultrasound has been the most frequently employed technique in European and Asian countries, while in USA CT and particularly CT fluoroscopy have been mostly used. Ultrasound is inexpensive, fast and widely accessible, but the evaluation of the ablation zone may be impaired by gas formation and patient’s physical conditions and the impossibility to precisely delineate the ablative margins. The (partially) limited sensitivity of ultrasound and its operator-dependent nature are counterbalanced by the use of contrast enhancement with microbubbles (contrast-enhanced ultrasound – CEUS) that enhances the target visibility for both HCCs and metastases and, particularly for the detection of hepatic metastases, allows to achieve a sensitivity similar to that of MRI with hepatospecific contrast agents.^64,65^ On the contrary, for the pre-treatment staging of HCCs, contrast-enhanced CT or MRI have greater sensitivity than CEUS. CT guidance allows to avoid the major limitations of ultrasound, but the differentiation between ablation zone and residual tumor is only possible for a very short time after administration of an intravenous contrast agent. In addition, CT guidance leads to considerable radiation exposures.

MRI guidance provides higher sensitivity than ultrasound and CT in depicting small parenchymal lesions in the pre-ablation and targeting phase both with standard sequences and DWI, and with gadolinium-based, liver-specific contrast agent administration. In addition, MRI allows to achieve non-enhanced and near real-time fluoroscopic imaging during the procedure with repeatable visualization of tumor tissue, surrounding anatomy, and ablation zone without the need of removing the ablation applicator, and accurate monitoring of thermal effects by MR-thermometry curves. The absence of ionizing radiation is a valuable advantage of MRI compared to CT, but the duration of MRI-guided procedures is usually longer than those with CT-guidance.^66^ In clinical studies comparing CT-guidance and MRI-guidance for ablation of HCCs and liver metastases^67,68^ and in a systematic review of the literature,^69^ technical success rates, LTP rates, and OS did not show significant differences, but reduction of the number of sessions required for complete tumor treatment^67^ and decreased number of complications^69^ for MRI guidance have been reported, even though combined with longer learning curve. Unfortunately, the use of MRI guidance is limited to specialized centers due to the restricted availability of MR scanners suitable for interventional procedures, and the high operating costs (MRI-compatible devices, etc..).

## Where are we now

In order to summarize the current situation of IO for the liver, also with a glance to the next few years’ developments, we have to take into account the five major topics: imaging techniques, the two main clinical applications (metastases and HCC), ablative/intravascular techniques, and combined/immunologic treatments.

### Imaging techniques

For precise localization and correct targeting of small or sonographically hardly visible hepatic lesions, several systems have been introduced in recent years and will be increasingly used in the future: fusion of real-time ultrasound with previously acquired CT, MRI or PET scans,^70–72^ coupling of fluoroscopy or real-time ultrasound and 3D imaging from C-arm cone-beam CT^73^ and stereotactic guidance with electromagnetic or optical tracking systems, assisted by aiming devices or robotic arms.^74,75^ Stereotactic or robotic guidance improves the accuracy of needle placement compared to conventional CT image guidance, especially when using off-plane trajectories.^76^ More recently, very fast and low cost systems of augmented reality (AR) guidance that allow easy and accurate 3D visualization of targets with significant reduction of the radiation dose compared to CT-guided ablation are being introduced in clinical practice.^77^

The precise ablation applicator placement is an important factor for successful ablation, in association with the conspicuity achieved. In recent years, it has been demonstrated that a concentric >5 mm ablative margin is a key factor to achieve a significant decrease of the local tumor progression (LTP) rate.^78–80^ In the immediate post-ablation phase, CEUS allows to assess the result being achieved and the possible presence of residual tumor, guiding supplementary targeted ablation,^64,65^ but the final modality to assess the technical success is conventionally the visual comparison of pre- and post-ablation contrast-enhanced cross-sectional images, *i.e.* the so-called side-by-side juxtaposition. This modality has, however, a high margin of error due to the misalignment of the liver due the patient’s position and the respiratory phases, and also to the tissue structural changes after ablation.^81^ To solve this problem, some software using the non-rigid registration of pre- and post-ablation CT or MR imaging have become available^82,83^ and their use will likely become mandatory for the precise assessment of the technical success of every percutaneous ablation.

In addition, PET enables to identify LTP following ablation of hepatic metastases earlier than intravenous contrast-enhanced CT and before morphological changes,^84^ and will be increasingly recommended for the assessment and the follow-up of patients with ablated hepatic metastases.

### Treatment of hepatic metastases

In spite of the many papers published on long-term results achieved in large number of patients,^80,85^ few guidelines have been produced, mostly for metastases from colorectal cancer and from National Societies.^86,87^ In 2015 a position paper by an international panel of ablation experts^78^ fixed some worldwide accepted statements, like the most commonly used cut-off maximal diameter (3 cm), the similar tumor control to hepatectomy alone when 3 metastases with size <3 cm are ablated with 10 mm or more margins,^88^ the complete eradication dependance on proper anatomical location and multiple overlapping ablations for metastases larger than 5 cm,^78^ the importance of the “test-of-time” proposed for the first time by Livraghi et al. in 2003,^89^ etc. In 2016, the European Society of Medical Oncology (ESMO) officially introduced thermal ablation, brachytherapy, SBRT, TACE and TARE in the therapeutic flow chart for colorectal hepatic metastases.^90^

Hepatectomy combined with intra- or post-operative ablation has been reported to achieve local tumor control and preserve the remaining liver in patients who have limited liver reserves and for recurrent or new tumor image-guided ablation can be used repeatedly with survival rates similar to patients without recurrence.^91^

For colorectal hepatic metastases, DEBIRI-TACE either with large or small particles^63,92^ and TARE associated with second-line chemotherapy^93^ are used as salvage therapies in selected cases with proven survival prolongation. On the contrary, for hypervascular metastases, mostly from neuroendocrine tumors, TACE and TARE have shown important long term efficacy.^94^ Few studies on thermal ablation of non-colorectal hepatic metastases have been published and most of them deal with metastases from gastric, breast, and pancreatic carcinomas^95–99^ because the indications for such treatments are limited: oligometastatic patients with liver-only metastases or with stable extrahepatic disease, inoperable and non-responsive to chemotherapy, or with recurrences after chemotherapy and/or surgery not amenable to further chemotherapy or resection. The most favorable characteristics of these treatments are the very low morbidity and mortality rates and the high technical success and local control rates if small size metastases are treated.

### Treatment of HCC

After the fundamental recognition of RFA as first-line treatment for “very early” HCC in operable patients,^100^ the role of ablation for the treatment of HCC of 5 cm or smaller, both primary and recurrent, has been definitely confirmed.^101^ In recent years, increasing importance is being given to patient’s liver function discussed in multidisciplinary meetings before selecting the best treatment, either local or systemic or both, for each HCC in each patient, because long-term outcomes have demonstrated that liver function and treatment outcome are strictly related.

Numerous guidelines for the management of HCC have been proposed by scientific Societies (EFSUMB, EORTC, ESMO, AASLD, APASL, etc.) and continuously updated, but the Barcelona Clinic Liver Cancer (BCLC), published for the first time in 2001, still remains the milestone. In the latest update,^102^ as major “novelties” compared to the previous versions, local expertise and technical availability are included among the important parameters for the management of HCC, and liver transplantation is indicated as one of the main therapies. Differences in etiology, screening modalities and medical care system account for some differences between Western and Asian guidelines, particularly for HCCs with vascular invasion: systemic chemotherapy and TARE are more recommended in Western countries^103^ and TACE in Asian countries.^104,105^

For selected early or intermediate stage HCC, TARE has similar complication rates and superior tumor control compared with TACE with drug-eluting beads^106^ and for early-stage HCC TARE segmentectomy achieves survival rates comparable to curative intent therapies, such as transplantation and surgical resection.

For advanced stage HCC with poor liver function, TACE as palliative treatment has been almost completely replaced by systemic treatments, such as the combination of programmed cell-death 1 ligand 1 (PD-L1) checkpoint inhibitor atezolizumab and bevacizumab, and the role of selective TACE has been progressively reinforced as complementary therapy to ablation in early stage HCC, *e.g*. for lesions not completely necrotized and/or with remnant vital tissue scattered or not recognizable at ultrasound examination for an additional ablation, and for satellite nodules after achievement of complete necrosis of the main HCC.

The most recent and promising developments of intravascular therapies for HCC are small drug-eluting microspheres (DEM-TACE)^107^ and balloon occlusion catheters (B-TACE),^108,109^ with outcomes still under evaluation.

### Ablative techniques

The advantages of MWA over RFA (lower rates of LTP and greater efficacy for perivascular tumors with satisfactory ablative margins) are well recognized, even though no significant difference for OS and ablation-related complications has been reported.^110^ In addition, also the technology of RFA is continuously improving, with outcomes approaching those achievable with MWA.^111^ For very large liver malignancies, stereotactic CT-guided ablation allows to currently achieve excellent technical success and long-term outcomes.^112^ For HCCs or metastases adjacent to large blood vessels and/or to central bile ducts, IRE, even though technically complex, can be almost safely used.^53–56^

Among the relatively new local ablative techniques, also high-dose-rate brachytherapy (HDR-BT) must be included. This technique, employed for both HCCs and liver metastases, uses a single-fraction irradiation, with an iridium-192-source directly placed in the target volume via percutaneously inserted catheters.^113,114^ Given its internal local approach, HDR-BT has no limitations regarding tumor size or proximity to vascular structures.

### Combined/immunologic treatments

In recent years, a lot of research has been carried out in combining percutaneous treatments with each other and/or with systemic treatments.^115^ In HCCs, the combination of TACE and thermal ablation leads to a reduction of the TACE-induced neo-angiogenesis, decreasing the risk of tumor recurrence, and increases the volume of coagulation reducing the LTP rate. Therefore, this combination can result particularly useful in patients unsuitable for resection and with large HCCs.^116^ The combination of TACE and stereotactic body radiotherapy (SBRT) can be also effective, given that SBRT works in tumor areas with high oxygenation (the periphery of HCC) and cytotoxic agents used for TACE give higher radiosensitivity.^117^ The combination of TACE and sorafenib and other tyrosine-kinase inhibitors did not provide promising results for HCCs, while the combination of locoregional treatments and immunotherapy seems to have all the prerequisites to be effective. In an environment with decreased proinflammatory cytokines and increased immunosuppressive cytokines (HCC), tumor necrosis induced by TACE and mostly by thermal ablation might lead to release of tumor-associated antigens, that could stimulate the specific immune response. This could enhance the effect of immunotherapy agents (such as regorafenib or nivolumab) programming the immune system against cancer cells. In addition, TACE-induced hypoxia increases the production of vascular endothelial growth factor (VEGF), which catalyzes recurrent tumor growth due to an increase in revascularization. VEGF inhibitors could, as counterpart, inhibit the revascularization.^118^

Also the combination of immunotherapy followed by ablation seems to provide valuable results. Ablation releases tumor-associated antigens that enhance the immune response against the tumor itself with stimulation to the release of specific CD8+T cells both for HCCs and hepatic metastases, but it also releases inflammatory cytokines that stimulate an antitumor systemic immune response, further enhanced when adjuvant immunotherapy is administered after ablation (thermal ablation as endogenous *in situ* tumor vaccination).^119–121^ This explains the phenomenon called “abscopal effect”, namely tumor regression in untreated lesions and inhibition to develop distant metastases, evidenced after distant thermal ablation. While it is not yet clear which ablative technique has the highest potential for releasing tumor antigens and creating the best immunostimulatory microenvironment,^119^ a new ablative technique (histotripsy) seems to have the highest capability to produce the abscopal effect.^122^ Generating short, high-amplitude micropulses of focused ultrasound, histotripsy allows to non-invasively treat tumors by cavitation, without thermal injury, inflammation and heat-sink effect and to spare collagen-based structures, like bile ducts, blood vessels, and glissonian capsule.^123^

In conclusion, immunotherapy has the potential to compensate various drawbacks of local or regional treatment modalities alone and to facilitate highly individualized treatment approaches in the context of personalized medicine, but further studies are needed to confirm the current initial results.
